# Strategies and prospects for biostimulants to alleviate abiotic stress in plants

**DOI:** 10.3389/fpls.2022.1024243

**Published:** 2022-12-22

**Authors:** Ying Ma, Helena Freitas, Maria Celeste Dias

**Affiliations:** Centre for Functional Ecology, Department of Life Sciences, University of Coimbra, Calçada Martim de Freitas, Coimbra, Portugal

**Keywords:** phytostimulants, plant beneficial microbes, abiotic stresses, climate change, modern agriculture

## Abstract

Global climate change-induced abiotic stresses (e.g., drought, salinity, extreme temperatures, heavy metals, and UV radiation) have destabilized the fragile agroecosystems and impaired plant performance and thereby reducing crop productivity and quality. Biostimulants, as a promising and eco-friendly approach, are widely used to address environmental concerns and fulfill the need for developing sustainable/modern agriculture. Current knowledge revealed that plant and animal derived stimulants (e.g., seaweeds and phytoextracts, humic substances, and protein hydrolysate) as well as microbial stimulants (e.g., plant beneficial bacteria or fungi) have great potential to elicit plant tolerance to various abiotic stresses and thus enhancing plant growth and performance-related parameters (such as root growth/diameter, flowering, nutrient use efficiency/translocation, soil water holding capacity, and microbial activity). However, to successfully implement biostimulant-based agriculture in the field under changing climate, the understanding of agricultural functions and action mechanism of biostimulants coping with various abiotic stresses at physicochemical, metabolic, and molecular levels is needed. Therefore, this review attempts to unravel the underlying mechanisms of action mediated by diverse biostimulants in relation to abiotic stress alleviation as well as to discuss the current challenges in their commercialization and implementation in agriculture under changing climate conditions.

## Introduction

Rapid climate change has exacerbated various biotic and abiotic stresses on agricultural plants/crops, causing a change in plants’ physiological, biochemical, cellular, and molecular mechanisms (Ma et al., 2020). The acceleration of global warming has devastating effects on plant growth and crop yield, as well as nutritional quality, threatening sustainable crop production worldwide (Saddiq et al., 2021). During the decades of the industrial revolution, humankind has relied on the massive use of chemical inputs to stimulate crop production, therefore continuously unbalancing its environment (Delitte et al., 2021). The intensive anthropogenic activities and rapid industrialization in combination with current climate change impacts have led to a reduction in the quality of arable land and environmental pollution (e.g., over-accumulation of heavy metals in the soils) (Oleńska et al., 2020). Under natural climate/field/agricultural conditions, plants usually face a combination of different environmental stresses such as drought, salinity, extreme temperatures, heavy metals, and UV radiation, contributing to the augmented joint stress severity (Ma et al., 2020; Srivastava et al., 2021). To adapt to these stresses, not all species have the ability to evolve their plastic responses and grow in such harsh environments (Shah et al., 2021). It was estimated by the U.S. National Climate Assessment that environmental stresses account for the losses in crop yield of up to 50%. In this regard, various agrochemical approaches have been developed to alleviate the adverse environmental effects and synthetic fertilizers (e.g., nano-silicon, FeO nanoparticles) and pesticides have been perceived as key agricultural inputs (Haghighi and Pessarakli, 2013; Manzoor et al., 2021; Silva et al., 2022). Nevertheless, while the imbalanced application of these agrichemicals enhances crop productivity, they also damage the environment and have impact on human health. There is an increasing demand for alternatives to traditional/conventional agrichemicals.

Lately, the application of biostimulants has been considered a potentially novel approach to stimulate plant growth and crop productivity under both stress and control conditions, representing a momentous/significant scientific breakthrough toward safeguarding future food production in a sustainable manner (Rai et al., 2021). Although numerous studies have demonstrated the promising potential, functions, and possible challenges of biostimulants to mitigate abiotic stresses and improve quality and yield (Van Oosten et al., 2017; Zaid et al., 2020), the underlying mechanisms of biostimulant-plant interactions at the cellular, metabolic and molecular levels to overcome stress adversities are still elusive (Nephali et al., 2020). In the light of recent advances in the field of plant-biostimulant interactions, this review attempts to unravel the underlying mechanisms of action mediated by diverse biostimulants in relation to abiotic stress alleviation as well as to discuss the current challenges in their commercialization and implementation in agriculture under changing climate conditions.

## Impact of abiotic stresses on plant performance

Global climate change scenarios have contributed to increasing the intensity of abiotic stresses, such as drought, salinity, heat, and high UV radiation, which lead to extensive losses in agriculture (Waqas et al., 2019). Moreover, climate change projections and crop yield models predict a worsening of losses in main agricultural crops, such as wheat, rice, and maize, in the next years, with serious impacts on food security (Tigchelaar et al., 2018). Therefore, the development of sustainable strategies to reduce the impact of abiotic stresses on plant performance is of high priority. Among the various strategies to mitigate the negative effects of abiotic stresses on plants, the use of eco-friendly compounds, such as microbial and non-microbial biostimulants, is one of the most promising (Rouphael et al., 2020; Ali et al., 2021).

As sessile organisms, plants developed sophisticated perception mechanisms, as well as signaling and acclimation strategies to cope with harsh environmental conditions, but at the cost of decreased growth and yield (Zandalinas et al., 2020). These mechanisms include increased production of antioxidant metabolites (e.g., flavonoids, proline, and enzymes) that help in the control of reactive oxygen species (ROS) to avoid oxidative stress and changes in the levels of phytohormones (e.g., ABA and JA) (Zandalinas et al., 2020; Ma et al., 2020; Dias et al., 2022; Dias et al., 2018). Hence, drought and salinity represent a major threat to plant productivity (Daliakopoulos et al., 2016; de Oliveira et al., 2022). The first morphophysiological responses of plants to drought and salinity are very similar, inducing water stress, leading to cellular dehydration and a reduction in water potential, hampering cell expansion and wall synthesis, growth and shoot development (Ma et al., 2020). Moreover, drought and salinity reduce transpiration and nutrient uptake (Ahluwalia et al., 2021). With the extent of drought, root elongation is continuous for groundwater search, while under salinity ionic stress also occurs, and roots started to accumulate a high quantity of ions, principally Na^2+^ (Ma et al., 2020). Photosynthesis impairment occurs, together with reductions in pigment synthesis and enzyme activities. Under these conditions, the light absorbed in excess and not used for photosynthesis can lead to oxidative stress (Ma et al., 2020). Some species have mechanisms of salt exclusion, and others concentrate salt in vacuoles (Khan et al., 2020), leading to lower negative effects on metabolic processes, such as photosynthesis. Also, some metabolites such as carbohydrates, amino acids, and nitrogen play a vital role in protecting plants against stresses by acting as osmolytes and/or ROS scavengers (Sharma et al., 2019).

Moreover, frequent and severe heat waves have been already occurring and affecting the phenology, productivity, and yield of many plants (Dusenge et al., 2019). Heat stress can increase membrane permeability, degradation of proteins and decrease in their synthesis, inactivation of enzymes, reduction of photosynthesis, and inhibition of pigment synthesis (Jagadish et al., 2021; Zhao et al., 2021). These effects can lead to a slowdown in growth and an increase in ROS production (Vargas et al., 2021).

In addition, plants are usually subjected to a simultaneous combination of different abiotic stresses, triggering specific responses (Savvides et al., 2016). Most of the studies reported higher negative impacts on plant performance when some abiotic stresses (drought and heat, salinity and heat) are combined, compared to each abiotic stress applied individually (Escobar-Bravo et al., 2017; Zandalinas et al., 2018; Zandalinas et al., 2020). For instance, in Arabidopsis, salinity combined with heat induces higher impacts on growth, chlorophyll content and Na^+^/K^+^ than these stresses applied separately (Suzuki et al., 2016). Dissimilarly, in tomato plants, heat combined with salinity improved photosynthesis, water and osmotic potential, and decreased protein oxidation and H_2_O_2_ levels when compared to the effects of salinity stress alone (Rivero et al., 2014). Plants seem to integrate simultaneously two different systemic signals generated when exposed to a combination of stresses, and the way plants perceive the stresses that trigger these signals strongly influences the intensity and efficiency of responses (ROS signals, changes in transcript abundance, hormonal, and stomatal responses) (Zandalinas et al., 2020). Moreover, these responses must be fast and well-coordinated between the distinct parts of the plants (leaves, roots, or stem) (Zandalinas et al., 2020).

## Biostimulants: Definition, type,and role in agriculture

Biostimulants are organic or inorganic compounds and/or microorganisms, that when applied to plants, stimulate several processes, leading to improved growth and productivity, and tolerance to stresses (Ali et al., 2021; Franzoni et al., 2022). They are available as soluble powder, granular form, or liquid and can be applied as foliar sprays or/and in soil near the root zone. Biostimulants are considered environmentally friendly agronomic tools, and the market for these compounds is increasing at an annual growth rate of 12% possibly reaching 5.6 billion dollars in 2026 (Bulgari et al., 2017; Lau et al., 2022). Several factors have influenced the increase in the biostimulant market, namely the changes in agricultural and environmental policies allied with climate change, pushed a necessity for more sustainable alternatives to synthetic chemicals (Lau et al., 2022). Biostimulants display low or null toxicity and do not accumulate in long term (Sangiorgio et al., 2020). There are several available types of biostimulants, and they can be classified based on the source of raw material into six major groups, such as seaweed, plant extracts, protein hydrolysates, humic substances, inorganic compounds and microorganisms (Franzoni et al., 2021).

### Seaweed and plant extracts

Biostimulants based on seaweeds are the most studied and commercialized (Cristiano et al., 2018). This group includes macroscopic and multicellular marine algae from several taxonomic groups, such as red, green, and brown algae (Bulgari et al., 2019). Seaweeds are used in agriculture as fertilizers since ancient times due to their positive effects on crops (Franzoni et al., 2022). These algae contain hormones (e.g., auxins and cytokinins), polyphenols (e.g., phloroglucinol and eckol), polysaccharides (e.g., fucoidan, laminaran, carrageenan and alginates) and kahydrin, a derivative of vitamin K1, that are growth promoters and activators of plant defense system (Ertani et al., 2018; Baltazar et al., 2021). Most of the products used in agriculture are based on the red (*Lithothamnium calcareum*, *Kappaphycus alvarezii*, *Jania rubens*, and *Gracilaria edulis*), green (*Ulva lactuca*) and brown (*Ascophyllum nodosum*, *Ecklonia maxima*, *Durvillaea potatorum* and *Laminaria*) algae (Van Oosten et al., 2017; Baltazar et al., 2021).

Some biostimulants can be obtained from extracts of different plant structures, leaves, fruits, stems, and flowers (Franzoni et al., 2022). These extracts are rich in bioactive compounds that can activate some physiological processes in plants, and thereby improve their performance (Zulfiqar et al., 2020). For example, root extracts of carrots and licorice have been shown to improve the growth of cowpea, beans, pear tree, and fennel. Also, leaf extracts from borage, aloe, garlic, and green tea improved growth and several physiological processes in various horticultural crops (such as lettuce, tomato, eggplants, guar, and olive trees) and ornamental plants (e.g., dwarf umbrella tree) (Ali et al., 2018; Elzaawely et al., 2018; Moreno-Hernández et al., 2020; Zulfiqar et al., 2020). Seagrass aqueous extracts mitigate the negative effects of salt stress in tomato plants, improving the activity of antioxidant enzymes (Vinoth et al., 2017). *Moringa oleifera* leaf extract is also used to increase plant growth in several crops, such as pumpkin, peas, and common bean (Del Buono, 2021). This beneficial action is associated with the high levels of minerals, carbohydrates, protein, amino acids, hormones, and antioxidant enzymes (Zulfiqar et al., 2020; Del Buono, 2021).

### Protein hydrolysates, humic substances and inorganic compounds

Hydrolyzed proteins are a mixture of amino acids, polypeptides, and oligopeptides obtained by chemical, thermal and enzymatic hydrolyses of proteins from different matrices, like animal or plant (e.g., plant-derived biomass or animal epithelial and connective tissues) (Cristofano et al., 2021). Protein hydrolysates derived from by-products of agricultural and industrial activities are gaining popularity as they represent a sustainable and eco-friendly solution to many wastes and contribute to a circular economy (Colla et al., 2017). Most of the commercialized protein hydrolysate biostimulants are from animal-derived proteins, and they have a higher nitrogen content (9 to 16% DM) and a slower release rate (Cristiano et al., 2018). The amino acids proline and glycine are the most abundant components in protein hydrolysate biostimulants based on collagen, while the glutamic acid is more abundant in biostimulants derived from plant sources (Baroccio et al., 2017).

Another kind of biostimulants are the humic substances (HS) that comprise humic and fulvic acids, and they are the main components of lignites, soil and peat (Van Oosten et al., 2017). Humic substances are supramolecular structures with hydrophilic (-OH and -COOH groups) and hydrophobic portions, resulting from the association of small molecules by the decomposition processes of animals, plants, or from the metabolic activity of soil microbes (Franzoni et al., 2022; Popa et al., 2022).

Some inorganic compounds such as silicon, selenium, cobalt, sodium, and aluminum can promote plant growth and induce stress responses, but they are not essential for all plants (Du Jardin, 2015). They can be found in plants and soils in different forms, as inorganic salts and insoluble, like the amorphous silica (Franzoni et al., 2022).

### Microorganisms

Microorganisms-based biostimulants are mainly comprised of bacteria, fungi, and arbuscular mycorrhizal fungi (AMF) that can be isolated from soils, plants, and organic materials (Baltazar et al., 2021; Del Buono, 2021). These biostimulants can be applied directly to the soils or seeds (Franzoni et al., 2021). These biostimulants interact directly with the plants, establishing a mutual symbiotic association or indirectly by improving nutrient availability to plants (Franzoni et al., 2021).

The plant growth-promoting bacteria (PGPB) include rhizobacteria, which colonize the rhizosphere, and free-living bacteria from the soil (Baltazar et al., 2021). The plant growth-promoting rhizobacteria (PGPR) are the most important group and include the genera *Azotobacter*, *Arthrobacter*, *Azospirillum*, *Acinetobacter*, *Bacillus*, *Bradyrhizobium*, *Enterobacter*, *Pseudomonas*, *Rhodococcus*, *Rhizobium*, *Streptomyces*, and *Ochrobactrum* (Rai et al., 2021). AMF can establish a symbiotic relationship with plants and are the most commonly used biostimulants (Du Jardin, 2015). This kind of biostimulant includes the *Rhizophagus* spp., *Septoglycus viscosum*, *Claroideoglomus etunicatum* and *Claroideoglomus claroideum* (Rai et al., 2021). Besides AMF, other fungal-based biostimulants are very used in sustainable agriculture production, such as the endophytic fungi *Trichoderma* spp. (Ascomycota) and *Sebacinales* (Basidiomycota) (Du Jardin, 2015).

## Mechanism of action of biostimulants for mitigation of abiotic stresses and plant growth

Although several studies indicated that they act as priming agents (Shukla et al., 2019), the action modes of microbial and non-microbial biostimulants in plants are not well known yet. Priming, or stress hardening, triggers several molecular and physiological defense mechanisms that increase plants’ ability to defend themselves when exposed to various stresses. These molecular and cellular changes induced by these priming agents in plants are stored as primed memory (Nephali et al., 2020) and once primed, the plants will defend faster and stronger to cope with subsequent stresses.

Biostimulants activate and regulate several defense mechanisms through different action modes (Rai et al., 2021). The biostimulants can be applied directly in the leaves (foliar application) or/and in the soil near the root system. The biostimulants based on proteins and amino acids (e.g., proline and glycine) can penetrate the leaf tissues directly and enter into the cells. The protein hydrolysates-based biostimulants enter the plant cell *via* diffusion processes through membrane pores, along with energy costs (Yakhin et al., 2017). Whereas in the microorganism-based biostimulants, the hyphae penetrate the tissues and establish symbiotic or mycorrhizal associations. When biostimulants reach the leaves and/or roots they are translocated and distributed to the other parts of the plant (Rai et al., 2021). Within the plant, the mechanisms of action differ according to the type of biostimulants (nature and characteristics of the biostimulants).

### Molecular, metabolic and physiological mechanisms

Several studies demonstrated that plant priming with microbial and/or non-microbial biostimulants results in enhanced plant defense response to stresses such as increased antioxidant enzymes activities, accumulation of polyphenols and osmolytes (Shukla et al., 2019; Vaseva et al., 2022). **Table 1** represents an overview of the molecular, metabolic, and physiological mechanisms underlying biostimulants-induced abiotic stress alleviation.

**Table 1:** Action mechanisms and beneficial effects of biostimulants (SE, seaweed; PH, protein hydrolysates; HS, humic substances; PGPB, plant growth promoting bacteria and AMF, arbuscular mycorrhizal fungi) in plants under drought, salinity, cold and heat stress.

Biostimulant	Stress	Action Mechanism	Beneficial effects	Reference
SE	Drought	Low expression of *MYB60* gene, increase of plant survival, water used efficiency (WUE), photochemical efficiency and non-photochemical quenching	Maintain the stomata conductance, improve water content and photosynthesis, and increase photoprotection	Shukla et al. (2019) Del Buono et al. (2021) Santaniello et al. (2017)
		Low expression of *NCED3*, *RD29* and *RAB18* genes	Lower water stress perception	
		Low expression of *RbCS1A* and *RCA* genes	Prevent the reduction of carboxylation capacity	
		Constant expression of *PIP1;2* and *βCAI* genes	Maintain the mesophyll conductance to CO_2_	
		Increase the expression levels of *DFR* and *SOD* genes	Enhance ROS scavenging processes	
		Increase fresh weight, dry weight, chlorophyll, proline and sugars, upregulate the expression of tas14 dehydrin gene, and reduce lipid peroxidation	Promote biomass production and growth, reduce membrane damages, and increase dehydration tolerance	Goñi et al. (2018)
		Increase the expression levels of *CYP707A1a*, *CYP707A3b* and *DREB2a*, *PIP* genes and enhance total antioxidant capacity	Reduce stomatal conductance, promote the antioxidant defense and drought tolerance	Shukla et al, (2018b); Shukla et al, 2019)
	Salinity	Increase biomass production	Promote plant growth	Jithesh et al (2019)
		Increase the expression of *LEA 4*, *RD29*, *Di21*, *RAB18, CCA1, LTP3 and LTP4* genes, and jasmonic acid levels	Enhanced salt tolerance	
		Increase the expression levels of *GOLS3* gene	Promote carbohydrate metabolism and growth	
		Upregulate the expression levels of *CHI*, *PAL1*, *PAL2*, *CHS, CHI*, *F3´H* and *DFR* genes	Increase the flavonoids biosynthesis	
		Increase root length and dry weight, carbohydrates, nutrients, proline, ascorbate and the expression levels of genes related to antioxidant enzymes (APX, SOD, DHAR and CAT)	Improve photosynthesis, promote growth and the antioxidant defense	Elansary et al (2016); Elansary et al (2017)
		Low expression of miR827, miR2111b, miR395 and miRNA399, and elicit the expression of glutathione S transferase	Improve nutrient uptake and antioxidant defense	Shukla et al. (2018a)
		Increase the expression of *SS*, *SPS*, *G6P*, *PGA*, *SUS*, *GLPT*, *SWEET 4* and *SWEET15, GOLS3, GOLS2*, xyloglucosyl transferase and *UDP* glucosyl transferase genes	Promote carbohydrate metabolism and cell wall biosynthesis	Del Buono et al. (2021) Shukla et al. (2019)
	Cold	Upregulate the expression of *P5CS1* and *P5CS2* genes	Increase proline synthesis and induce osmoregulation	Nair et al. (2012)
		Increase in the transcription of genes *COR15A* and *RD29A*, and the transcription factor CBF3	Protect membrane integrity (stabilize chloroplast membranes)	Ali et al. (2021)
		Upregulate the expression of *GOLS2* and *GOLS3* genes	Increase carbohydrate metabolism and induce osmoregulation	
PH	Drought	Activation of antioxidant enzymes activities (SOD and APX)	Increase ROS scavenger	Agliassa et al. (2021)
		Increase the levels of salicylic acid, carotenoids, hydroxycinnamic amide signaling, and prenyl quinone radical scavenging	Enhance stress tolerance	Paul et al. (2019)
	Salinity	Upregulate the expression levels of *PAL*, and increase the levels of flavonoids, terpenes, carbohydrates, carotenoids, salicylic acid, sterols and amino acids	Enhance antioxidant defense and stress tolerance, induce osmoregulation	Ertani et al (2013) Lucini et al (2015)
		Enhance seed yield and plant biomass	Increase fiber production	Di Mola et al. (2021)
		Stimulate GA and GOGAT enzyme activities	Increase protein content, photosynthesis, and growth	Ertani et al. (2013)
	Heat	Overexpression of HSP genes (*HSP16.9*, *HSP22*, *HSP16.9* and *HSP116.9.*) and DHN transcripts (DHN1, DHN2, DHN4, DHN13, DHNXero1 and DHNCor410)	Enhance stress tolerance: increase membrane stabilization	Vaseva et al. (2022)
		Increase the levels of chlorophylls and carotenoids	Improve photosynthesis	Botta (2013)
HS	Drought	Increase plant dry weight, shoot fresh weight and leaf area, enhance nutrient uptake, chlorophyll content and promote the activity of antioxidant enzymes (SOD, CAT and Gr)	Increase plant growth and antioxidant defense	Kıran et al, 2019
		Increase nitrogen content, water potential and relative water content	Increase plant water status and plant biomass	Aguiar et al. (2016)
		Stimulate the activity of the antioxidant enzyme POD, and increase the levels of ABA	Increase antioxidant defence	García et al (2014)
		Enhance the activity of antioxidant enzymes (SOD and CAT) and increase protein and proline levels	Induce osmoregulation and increase antioxidant defense	Shen et al. (2020)
		Enhance anthocyanins levels	Enhance antioxidant defense	Sanjarimijani et al. (2016)
		Increase fresh and dry matter of roots and aboveground	Increase plant biomass and growth	Matuszak-Slamani et al. (2022)
		Increase chlorophyll levels, gas exchange and electron transport rate	Promote photosynthesis	Lotfi et al. (2018)
	Drought + Salinity	Enhance the activity of antioxidant enzymes (SOD, POD and CAT), increase nutrient uptake and chlorophyll production, and decrease electrolyte leakage	Increase antioxidant response and reduce membrane damages, improve nutrient availability and photosynthesis	Alsamadany (2022)
	Salinity	Activate antioxidant enzyme activities (SOD, CAT, GR and APX) and increase the production of antioxidants (tocopherol, ascorbate and phenolics)	Reduce oxidative stress	Aydin et al. (2012)
		Reduction of H_2_O_2_, electrolyte leakage and lipid peroxidation, and increase fresh weight	Reduce membrane damages and enhance biomass	Kaya et al. (2018)
		Increase root plasma membrane H^+^-ATPase activity, chlorophyll production, and enhance the metabolism of nitrate, TCA cycle, and phenylpropanoids	Increase nutrient uptake, growth and antioxidant defense	Nephali et al. (2020)
		Increase shoot fresh and dry weight and nutrient uptake	Enhance nutrient availability and plant growth	Çimrin et al. (2010)
	Heat	Overexpression of HSP genes (*HSP101*, *HSP81.8* and *HSP17.6A*)	Increase thermotolerance: protecting proteins denaturation	Cha et al. (2020)
PGPB	Drought	Increase nutrient uptake, 1-aminocyclopropane-1-carboxylate (ACC) deaminase, exopolysaccharides, chlorophyll content, the activity of antioxidant enzymes (SOD, CAT, POX, APX and GR), siderophore production, phytohormones (e.g., ABA, GA, IAA and cytokinins) and induce osmolyte accumulation (e.g., proline)	Enhancement nutrients availability, promote growth, and increase the antioxidant defense	Kaushal and Wani (2016)
		Increase root and sooth length, dry mass, upregulate the expression of genes *Cadhn*, *VA*, *sHSP*, *CaPR-10*, and induce the production of IAA	Increase plant growth, and drought tolerance	Lim and Kim (2013)
		Overexpression of trehalose-6-phosphate synthase and *P5CS* genes	Improve drought stress tolerance	Etesami and Maheshwari (2018)
		Increase root and shoot length, dry weight, water content, ACC deaminase and the activity of SOD, POX and CAT, and upregulate *DREB2A*, *CAT1*, *DHN* genes,	Enhance growth and stress tolerance	SarmaSaikia et al (2014)
	Salinity	Induce the production of IAA, increase fresh and dry weight, plant height, increased the expression of *DREB2b*, *RD29A*, *RD29B*, *RAB18*, *P5CS1* and *P5CS2*, *MPK3* and *MPK6*, increase total antioxidant capacity, and increase ACC deaminase	Increase growth, induce osmoregulation and enhance salt stress tolerance	Kim et al. (2014)
		Increase fresh and dry weight accumulation, leaf number, and leaf area, promote water and nitrogen use efficiency	Increase biomass and growth, improve plant water status	Miceli et al. (2021)
		Increase ACC deaminase, increase nutrient uptake, induce osmolyte accumulation by inducing the production of compatible organic solutes (e.g., glycine betaine and proline), increase nitrate reductase, chlorophyll and polyphenols levels, enhance the activity of antioxidant enzymes (SOD, CAT, POX APX and GR), increase the levels of IAA and gibberellins.	Enhance nutrient availability, ion homeostasis osmoregulation, and antioxidant defense	Kaushal and Wani (2016)
AMF	Drought	Increase root and shoot fresh weight, shoot length, and upregulate the expression of genes related to cellulose biosynthesis and cell growth (*XM_020312442.1*, *XM_020331230.1* and *XM_020312442.1*)	Increase growth and strengthen cell wall	Tarnabi et al. (2020)
		Increase water retention, photosynthesis, and water use efficiency	Improve photosynthesis, plant growth and water status	Oliveira et al. (2022)
		Promote plant biomass production, gas-exchange and WUE, increase the activity of SOD and reduce the levels of lipid peroxidation	Increase growth, and reduce oxidative stress (membrane protection)	Li et al. (2019)
	Salt	Increase biomass production, soluble sugars, protein, and organic acids	Enhance growth and antioxidant defense	Sheng et al. (2011)
		Increase sugars, compatible solutes (proline and glycine betaine), WUE, chlorophyll, ABA levels and nutrients uptake, enhance the activity of antioxidant enzymes, and reduce cell membrane permeability	Increase growth, nutrient availability, osmoregulation and reduce oxidative stress	Latef and Miransari (2014)
	Heat	Enhance total antioxidant activity, antioxidant enzymes activity (e.g., SOD and APX) and antioxidants (e.g., ascorbate and total phenols), and improve root water absorption	Reduce ROS accumulation, promote photosynthesis and increase water availability	Maya and Matsubara (2013) Mathur and Jajoo (2020)

### Seaweed

The seaweed extracts, as one of the most used biostimulants, can be used to increase plant tolerance to various abiotic stresses (Shukla et al., 2019; Ali et al., 2021). Plant treatment with seaweed-based biostimulants can act in central metabolic pathways, involved in photosynthesis, activating the defense system, and increasing ROS scavenging levels (Shukla et al., 2019). Moreover, biostimulants based on *A. nodosum* promote growth, increasing Arabidopsis length, tomato plants fresh and dry weight, and seashore paspalum root length and dry weight (Elansary et al., 2017; Goñi et al., 2018; Shukla et al., 2018a; Jithesh et al., 2019). Concerning photosynthesis, several improvements achieved under stress conditions were related to seaweed priming. For instance, seaweed biostimulants based on *A. nodosum* are described to regulate stomatal conductance in Arabidopsis by modulating the expression of *MYB60*, *NCED3*, *RK2*, aquaporins (*PIP1;2*) and *bCA1* genes and increase dehydration protection by activating the expression of several late embryogenesis abundant proteins (LEA, from group 2) and dehydrins genes (such as *RAB18*) under drought and salinity stress (Santaniello et al., 2017; Shukla et al., 2019). These changes induced by these biostimulants enable the plants to preserve water status and avoid dehydration, increase photosynthesis and water use efficiency (WUE), and protect proteins and enzymes under drought and salt stress conditions (Del Buono, 2021). Seaweed extracts of *A. nodosum* modulate the expression of genes involved in the biosynthesis of abscisic acid (ABA) (an increase of NCED3) in *Glycine max* L., resulting in less partial stomatal closure and higher WUE, and also in the catabolism of ABA by regulating the expression of CYP707A1a and CYP707A3b under drought stress (Shukla et al., 2018b). Moreover, other ABA-dependent genes, like *Di-21*, and lipid transfer protein (*LTP3* and *LTP4*) are positively regulated by seaweed biostimulants extracts of *A. nodosum*, thus enhancing Arabidopsis tolerance to oxidative stress under salt conditions (Jithesh et al., 2019). Other phytohormones, like jasmonic acid, with an important role in plant stress defense, are also positively regulated by seaweed extracts (Jithesh et al., 2019).

Moreover, extracts from *A. nodosum* act directly at the carbon assimilation levels by adjusting the expression of *RbCS1A* and *RCA* genes, which are related to RuBisCO activation (Santaniello et al., 2017; González-Morales et al., 2021), protect the photosystem II through the regulation of the *FIB1a* expression, promote the biosynthesis carbohydrates such as sucrose, raffinose and starch (increase the expression of *raffinose synthase (RS)*, *GOLS2* and *GOLS3*, *LHCB4*, *SS*, *SPS*, *G6P*, *PGA*, *SUS*, *GLPT* and *SWEET 4* and *SWEET15*), sugar alcohols (e.g. trehalose and myo-inositol), amino acids (e.g. proline and isoleucine), pigments and proteins, therefore increasing salinity, drought and low-temperature stress tolerance in Arabidopsis, tomato plants, wheat and sweet pepper (Goñi et al., 2018; Ali et al., 2021). The increased chlorophyll contents are linked to the upregulation of choline monooxygenase (CMO) and betaine aldehyde dehydrogenase (BADH), and also to the activation of protective mechanisms that reduce pigments degradation (Ali et al., 2021). Seaweed biostimulants based on *A. nodosum* positively regulate the expression of *P5CS1* and *P5CS2* genes linked to the biosynthesis of proline, and negatively regulate the expression of other genes related to proline catabolism in Arabidopssis (Rai et al., 2021), increasing plant tolerance to several types of abiotic stresses. Seaweed extracts (*A. nodosum*, *K. alvarezii*, *E. maxima* and *Laminaria* spp.) also improve nutrient uptake in maize, cottonwood, and mustard plants under drought and salinity stress (Shukla et al., 2019; Ali et al., 2021). Transcriptomic analysis indicated that *A. nodosum* extracts could increase S and P uptake under salt stress by modulating the expression of *miR827*, *miR2111b*, *miR395* and *miRNA399* in *A. thaliana* (Shukla et al., 2018a).

The mechanism of ROS scavenging activated by the seaweed involves both enzymatic antioxidants and non-enzymatic antioxidants. Seaweed biostimulants based on *A. nodosum* increase the activity of the antioxidant enzymes (e.g., CAT, SOD, and APX), and the production of antioxidants (e.g., ascorbate), leading to lower ROS accumulation and less membrane damage in plants (e.g., *Phaseolus vulgaris* and *Paspalum vaginatum*) under drought and salt stress (Elansary et al., 2016; Elansary et al., 2017; Shukla et al., 2019). Moreover, they down-regulate the expression levels of the GROWTH-REGULATING FACTOR7 (*GRF7*) gene resulting in an overexpression of the genes *DREB2a* and *RD29* and interact with several transcription factors like *COR47*, *COR15A*, *CCA1*, *NF-YA*, *AGF2* and *LHY1* that provide drought stress tolerance in *G. max* (Shukla et al., 2018b). *A. nodosum* extracts alleviate the oxidative stress levels under salinity by eliciting the expression of glutathione S transferase, regulating the ath-miR398 that modulates the expression of the copper/zinc SOD (*CSD1*) gene (Shukla et al., 2018a) and stimulating the phenylpropanoid pathway and flavonoids biosynthesis, by increasing the expression of ammonia lyase 1 and 2 (*PAL1* and *PAL2*), chalcone synthase (*CHS*) and isomerase (*CHI*), flavonoid 3´-hydroxylase (*F3´H*) and dihydroflavonol 4-reductase (*DFR*) genes in Arabidopsis (Jithesh et al., 2019). The glutathione S transferase also participates in the transport of flavonoids to vacuoles by ABC transporters, whose expression is induced by seaweeds (Jithesh et al., 2019).

### Protein hydrolysates

The mode of action of PH involves activation of the antioxidant system and improvement of photosynthesis (Lucini et al., 2015; Elansary et al., 2017). Plant-derived PH (alfa-alfa derived protein hydrolysate and LISIVEG^®^) upregulate the expression levels of phenylalanine ammonia lyase (PAL) and induce the production of defense-related metabolites such as flavonoids, terpenes, carbohydrates, sterols, and amino acids to increase salt stress tolerance in lettuce and maize plants (Ertani et al., 2013; Lucini et al., 2015). Under drought conditions, plant-derived PH (GHI_16_VHL and Trainer^®^) improved tolerance to ROS through a coordinated action of salicylic acid, carotenoids, hydroxycinnamic amide signaling, and prenyl quinone radical scavenging (Paul et al., 2019) and/or by the activation of antioxidant enzyme (e.g., SOD and APX) in *Solanum lycopersicum* and *Capsicum annuum* (Agliassa et al., 2021). PH (LISIVEG^®^ and GHI_16_VHL) also promote osmoregulation to increase tolerance to drought and salinity in *C. annuum* and *Lactuca satuca* (Lucini et al., 2015; Agliassa et al., 2021). Moreover, these plant-derived biostimulants (alfa-alfa derived protein hydrolysate and Kaishi^®^) act at the photosynthesis and growth level by increasing protein production, glutamine synthetase (GS) and glutamate synthase (GOGAT) activities to improve salinity tolerance (Ertani et al., 2013), and protein degradation protection and starch maintenance for thermotolerance in *Zea mays* (Vaseva et al., 2022). Under salinity stress, the animal-derived PH Stressal is reported to lower chloride uptake and translocation to aerial parts, therefore reducing leaf necrosis symptoms in *Diospyros kaki*  (Visconti et al., 2015). Colla et al. (2015) and Carillo et al. (2019) reported for maize and spinate that the protective effect on the photosynthetic apparatus under drought conditions was due to an auxin-like activity effect of the PH (Trainer^®^). Also, a plant-derived PH (Terra-Sorb^®^ Foliar) improve the photosynthetic efficiency under heat conditions, by upregulating the levels of chlorophylls and carotenoids in *L. sativa* and *Lolium perenne* (Botta, 2013). PH (Kaishi^®^)) induced the overexpression of several heat shock proteins (HSP), like *HSP16.9* and *HSP22* in leaves and *HSP16.9* and *HSP116.9* in roots, providing higher heat tolerance to maize through membrane stabilization (Vaseva et al., 2022). Furthermore, PH upregulate dehydrins (DHN) transcripts (DHN2, DHN4, DHN13, DHNXero1, and DHNCor410) in leaves and in roots (DHN1 and DHN4) under heat stress, thus increasing protection from protein denaturation and membrane fluidity (Vaseva et al., 2022).

The positive effects of these kinds of biostimulants under salinity stress are also visible in the production, boosting seed yield and plant residual biomass of *Cannabis sativa*, which is relevant in fiber production (Di Mola et al., 2021).

### Humic substances

Concerning the HS, they trigger several molecular processes that lead to physiological responses in plants, conferring tolerance to several types of stresses. HS can induce overexpression of several families of HSP, such as *HSP101*, *HSP81.8*, and *HSP17.6A*, increasing Arabidopsis thermotolerance by helping in repairing processes of denatured proteins and in maintaining protein homeostasis (Cha et al., 2020). Moreover, HS (e.g., extracted from leonardites and vermicompost) stimulate nutrient uptake (N, P, K, Ca, Fe, Mg, S, Mn, and Cu), root plasma membrane H^+^-ATPase activity, chlorophyll production, and enhance the metabolism of nitrate, TCA cycle, and phenylpropanoids in several species (e.g., lettuce, bean, garden cress, maize and sweet pepper) under salt stress (Çimrin et al., 2010; Aydin et al., 2012; Kaya et al., 2018; Nephali et al., 2020). The application of HS (K-humate-Proxin 85) increases salinity tolerance by decreasing oxidative stress through the reduction of the levels of ROS (e.g., H_2_O_2_), MDA and cell membrane permeability in beans and maize plants (Aydin et al., 2012; Kaya et al., 2018). These benefits lead to maize biomass increase and salt stress tolerance (Kaya et al., 2018). Under drought and salinity conditions, nutrient uptake is also improved, and Mg increase promotes chlorophyll production, leading to higher photosynthesis in *Vigna radiata* (Alsamadany, 2022). The upregulation of drought stress tolerance genes, *DREB2A*, *bZIP17* and *HsfA6a* is also modulated by HS application (Alsamadany, 2022). Moreover, HS (e.g., derived from vermicompost) prevent drought and salinity-induced oxidative stress by enhancing antioxidant enzymes activities (SOD, CAT, Gr, and APX), and osmoprotectants (proline) that reduce ROS levels in several species (e.g., *Oryza sativa*, *Saccharum officinarum*, *Cucumis melo*, *Setaria italica* and *Vigna radiata*) (García et al., 2014; Aguiar et al., 2016; Kıran et al., 2019; Shen et al., 2020; Alsamadany, 2022). HA exerted a drought stress-protective effect in *O. sativa* through a signaling mechanism independent of ABA, regulating the expression of tonoplast aquaporin (TIPs) genes, putatively triggered by chemical and physical interactions between HA and the root system (García et al., 2014). These biostimulants substances can also promote the plant water status by increasing the relative water content and water potential, as well as increase N content leading to higher biomass accumulation in *S. officinarum* and *C. melo* plants under drought conditions (Aguiar et al., 2016; Kıran et al., 2019). Matuszak-Slamani et al. (2022) also reported that HS (e.g., derived from vermicompost) increases several other morphological features in soybean, like fresh and plant dry matter of root and aboveground, and plant length under drought conditions. Furthermore, HS improve photosynthesis under water deficit conditions by stimulating gas exchange, electron transport rate, and chlorophyll content in *Brassica napus* (Lotfi et al., 2018), and promote vegetative and generative yield, as well as anthocyanins accumulation in *Hibiscus sabdariffa* (Sanjarimijani et al., 2016). Signaling pathways of several hormones such as ABA, gibberellins and auxins, and stress-responsive genes are positively modulated by HS, therefore alleviating salinity, drought, and heavy metal adverse effects in maize (Canellas et al., 2020).

### Microorganisms

The beneficial effects of several bacterial and fungal strains on plant growth under stress conditions have been studied (Etesami and Maheshwari, 2018; Leontidou et al., 2020). Arbuscular mycorrhizal fungi (AMF) and plant growth-promoting bacteria (PGPB) are the most common microbial biostimulants that act in stimulating plant performance (Miceli et al., 2021).

Several PGPB, such as *Azospirillum* sp., *Achromobacter* sp., *Aeromonas* sp., *Acetobacter* sp., *Bacillus* sp., *Bradyrhizobium* sp., *Chryseobacterium* sp., *Flavobacterium* sp., *Sinorhizobium* sp. and *Pseudomonas* sp. promote plant growth and development under stress conditions, leading to higher crop yield (e.g., *Arabidopsis thaliana*, *Pinus halepensis*, *Cucumis sativus*, *Oryza sativa* and *Phyllobacterium brassicacearum*) (Kaushal and Wani, 2016). PGPB are able to enhance plant tolerance to drought and salinity through various mechanisms including: a) synthesis of the enzyme 1-aminocyclopropane-1-carboxylate (ACC) deaminase that promote plant growth by cleaving plant-produced ACC, and thereby lowering the ethylene level in plants; b) enhancement of availability of nutrients (e.g. N, P, K, Fe, Mn, Zn, Cu and B) by solubilization and mineralization (e.g., N fixation, P solubilization, and siderophores production); c) osmolyte accumulation by inducing the production of soluble sugars and compatible organic solutes (e.g., proline and glycine betaine); d) production of phytohormones (e.g., ABA, GA, IAA and cytokinins); e) production of volatile organic compounds by modulating the expression of genes related to cell wall structure, synthesis of choline and glycine betaine, and stomata closure; f) decrease oxidative stress through the scavenging of ROS mediated by antioxidant enzymes (SOD, CAT, APX and Gr) and antioxidant metabolites (ascorbate and phenolic compounds); and g) maintenance of ion homeostasis by increasing K^+^ and sustaining K^+^/N^+^ ratio (Hayat et al., 2010; Kim et al., 2014; Kaushal and Wani, 2016; Ertani et al., 2018; Leontidou et al., 2020; Miceli et al., 2021). Microarray analysis indicated that *Rhizobium tropici* and *Paenibacillus polymyxa* induce the overexpression of the trehalose-6-phosphate synthase (TPS) gene involved in *P. vulgaris* drought tolerance (Ertani et al., 2018). Also, *Bacillus* strains upregulate the expression of the *P5CS* genes involved in proline biosynthesis, leading to osmotic tolerance in maize and cucumber plants (Ertani et al., 2018). *Pseudomonas aeruginosa*, *Paenibacillus polymyxa*, and *Bacillus licheniformi* activate the expression of the genes *ERD15*, *sHSP*, *CaPR-10*, *VA* and *Cadhn*, conferring drought tolerance to pepper plants (Lim and Kim, 2013).

Plant symbiotic association with several AMF strains (e.g., *Glomus* spp, *Rhizophagus* spp, and *Funneliformis* spp) are described to improve several physiological processes in plants (e.g., wheat, barley, maize, soybean, strawberry, onion and olive tree), therefore increasing plant tolerance to stresses (Begum et al., 2019; Oliveira et al., 2022). The mechanisms by which AMF can enhance plant tolerance to drought and salinity involve a) oxidative stress mitigation through the upregulation of antioxidant enzymes (SOD and CAT) and metabolites (glutathione), and downregulation of lipoxygenase; b) improvement of nutrient availability (e.g. Mg^2+^ and N) through the facilitation of uptake and modifications of the root morphology; e) stomatal regulation by controlling ABA metabolism; f) increase of photosynthesis, chlorophyll production, leaf water relation and photosynthate translocation leading to better growth and plant water status; g) osmolyte accumulation by inducing the production of compatible organic solutes (e.g., proline and betaine); and h) adjust the levels of growth regulators (cytokinins) (Sheng et al., 2011; Latef and Miransari, 2014; Li et al., 2019; Begum et al., 2019; Diagne et al., 2020; Oliveira et al., 2022). The AMF *Funneliformis mosseae* modulate the expression of *XM_020312442.1*, *XM_020331230.1*, *XM_020312442.1* genes related to cellulose biosynthetic process and cell growth, promoting drought tolerance in *Triticum aestivum via* strengthening cell wall and membrane (Tarnabi et al., 2020). Another AMF, like *Funneliformis* (*Glomus* spp.), can increase plant thermotolerance, through the enhancement of the total antioxidant activity, antioxidant enzymes activity (e.g., SOD and APX) and antioxidants (e.g., ascorbate and total phenols) that reduce ROS accumulation, and by helping *Cyclamen persicum* and *Z. mays* plants to improve water absorption to ensure higher photosynthetic capacity (Maya and Matsubara, 2013; Mathur and Jajoo, 2020).

Evidently, microorganisms play an essential role in environmental stress tolerance/resistance. However, designing and developing efficient commercial formulations of microbial inoculants has been the key factor determining the successful implementation of these beneficial microbes in agriculture (e.g., microbes could survive in the formulation products) (Bashan et al., 2014; Ma et al., 2020). Many bacterial genera such as *Azotobacter*, *Azospirillum*, *Rhizobium*, *Bacillus*, and *Pseudomonas* isolated from alkaline, saline, arid and acidic soils are found to have great potential to adapt to such adverse conditions and mitigate plant abiotic stress responses (in species like maize, wheat, pea, fava bean, chickpea, cotton, sorghum, potatoes, lettuce and sweet pepper), which can be served as biostimulants (Van Oosten et al., 2014). For instance, inoculation of certain microbial biostimulants *via* soil/seedling drench and seed coating is able to change plant cell wall composition by producing exopolysaccharides to form a protective biofilm on the root surface and accumulate high concentrations of solutes by synthesizing phytohormones (Egamberdieva et al., 2017), therefore increasing water retention and/or tolerance to salinity-induced ionic and osmotic stress in chickpea plants. The beneficial and protective functions of biostimulants on plants under diverse environmental stresses such as drought, salinity, and extreme temperatures are of vital importance for crops to survive in such environments. However, the development and selection of biostimulants should consider the physiological, biochemical, metabolic, and genetic mechanisms to cope with various stresses.

## Implementation of biostimulants in agriculture under climatic stress

Biostimulants were initially used in France, Italy, and Spain, and were later extended to other EU countries, America, Australia, etc., to improve agricultural production and quality worldwide, particularly under climate change-induced abiotic stress (Li et al., 2022). In Europe, biostimulants have been used in many types of plants and crops, such as vegetable crops (e.g., *Allium cepa, Capsicum annuum, Brassica oleracea, Solanum tuberosum, Cucumis sativus, Allium sativum, Solanum lycopersicum*, *Cucurbita pepo*, *Daucus carota*, *L.* sativa, and *Solanum melongena*), fruit crops (e.g., *Olea europaea*, *Prunus* sp., *Fragaria ananassa*, *Cucumis melo*, *Citrullus lanatus*, *Vitis vinifera*, *Citrus sinensis* and *Pyrus communis*), grain crops (e.g., *Hordeum vulgare*, *Triticum aestivum*, *Oryza sativa*, and *Zea mays*), oil crops (e.g., *Brassica napus* and *Glycine max*), horticultural crops (e.g., flowers, nurseries, lawns), and ornamental plants. Biostimulants have been widely used at all stages of production of the above-mentioned crops under various climatic stresses including as seed treatments, as foliar sprays during growth, as well as on harvested products (Khan et al., 2020; El Boukhari et al., 2021; Sorrentino et al., 2021; Jacomassi et al., 2022).

Biostimulants have been shown to affect multiple metabolic processes in crops, such as respiration, photosynthesis, ion transport, redox reactions, DNA synthesis, etc. (Rai et al., 2021). Numerous studies and practices have shown that biostimulants are capable of improving the ability of crops to absorb and utilize mineral nutrients, the utilization rate of fertilizers and crop health, plant resistance to various stresses, and ultimately achieve enhanced crop yield and quality products. **Table 2** presents the categorized biostimulants and their action mechanism and implementation in crop performance.

**Table 2:** From lab to field implementation of biostimulants in crop production.

Biostimulants		Abiotic stress	Crop species	Growth conditions	Effects on crop performance	Reference
Seaweed extract	*Ascophyllum nodosum*	Drought	*Glycine max*	Growth chamber	Over-expression of drought-responsive transcription factors	Shukla et al. (2018a)
	*A. nodosum*	Drought	*G. max*	Pot experiments	Improve plant drought tolerance by changing physiology and gene expression	Shukla et al. (2018b)
	*A. nodosum*	Drought	*Saccharum* spp. Hybrids	Field experiments	Alleviate drought stress while enhancing sugarcane development, stalk yield, sugar production, and plant physiological and enzymatic processes	Jacomassi et al. (2022)
	*A. nodosum*	Drought	*Spinacia oleracea*	Pot experiments	Improve leaf growth by enhancing leaf water relations, reduce ferrous ion chelating ability	Xu and Leskovar (2015)
	*Sargassum latifolium*, *Ulva lactuca* and their mixture	Drought	*Triticum aestivum*	Pot experiments	Antagonize oxidative damaging effects of drought by activating antioxidative systems and providing hormones and micronutrients.	Kasim et al. (2015)
	*U. lactuca*	Salinity	*Solanum lycopersicum*	Pot experiments	Attenuate the negative effects of salinity on plants	El Boukhari et al. (2021)
	*Zostera marina*	Salinity	*S. lycopersicum*	Pot experiments	Resistance to *Fusarium udum* and salinity by enhancing expression of stress responsive *CcWRKY* genes	Vinoth et al. (2017)
	*A. nodosum, Laminaria digitata*	Drought	*S. lycopersicum*	Pot experiments	Mitigate water stress effects by decreasing ABA, MDA and proline, increasing stem water potential and photosynthetic pigment levels	Campobenedetto et al. (2021)
Protein hydrolysates	Protein hydrolysates	Salinity	*Zea mays*	Pot experiments	Stimulate nitrogen metabolism, antioxidant systems, and plant biomass	Ertani et al. (2013)
	Protein hydrolysates	Salinity and cold	*Lactuca sativa*	Pot experiments	Improved crop tolerance to salinity, nitrogen metabolism and PSII efficiency (F_v_/F_m_)	Lucini et al. (2015)
	Protein hydrolysates	Salinity	*Diospyros lotus*	Field experiments	Decrease chloride uptake, stimulate chloride exclusion and osmolytes biosynthesis	Visconti et al. (2015)
	Protein hydrolysates	Drought	*S. lycopersicum*	Pot experiments	Stimulate action of signaling compounds, radical scavengers, reduce biosynthesis of tetrapyrrole coproporphyrins, thus improving tolerance to ROS-mediated oxidative imbalance	Paul et al. (2019)
	Protein hydrolysates	Salinity	*Arabidopsis thaliana*	Plate experiments	Reduce contents of stress-related molecules (e.g., flavonoids and terpenoids) and phytohormones (e.g., cytokinins, auxins, gibberellins), thus alleviating salt stress	Sorrentino et al. (2021)
	Protein compounds	Heat	*Lolium perenne*	Pot experiments	Improve fresh weight, stomatal conductance, photosynthetic efficiency, chlorophylls and carotenoids	Botta (2013)
Humic substances	Humic acids	Acidity	*Zea mays*	Petri dish	Triger weak acid stress response in cells, inducing plant acclimation and enhancing abiotic stress tolerance	Baía et al. (2020)
	Humic acids	Drought	*Oryza sativa*	Growth chambers	Activate antioxidative enzymatic function, thus controlling ROS content and modifying *OsTIP* expression	García et al. (2014)
	Humic acids	Drought, salinity	*Z. mays*	Environment cabinets	Exudation yield induced by humic acids enhanced the release of chemical compounds to root interface	Canellas et al. (2019)
	Humic acids	Salinity	*Phaseolus vulgaris*	Pot experiments	Increase plant nitrate, nitrogen and phosphorus and plant growth parameters, reduce soil electricity conductivity, proline and electrolyte leakage	Aydin et al. (2012)
Microorganisms	*Azospirillum brasilense*	Salinity	*L. sativa*, *Vicia faba*, *T. aestivum*	Pot experiments	Enhance plant growth, product quality, storage life and chlorophyll content	Fasciglione et al. (2015)
	*Pseudomonas frederiksbergensis*	Cold	*S. lycopersicum*	Field experiments	Confer stress tolerance, improve germination, plant growth and induce antioxidant capacity	Subramanian et al. (2016)
	*Glomus iranicum*	Salinity	*Euonymus japonica*	Pot experiments	Improve plant growth by increasing mineral nutrition (P, Ca and K)	Gómez-Bellot et al. (2015)
	*Pseudomonas fluorescens*	Salinity	*Cajanus cajan*	Field experiments	Confer resistance to *Fusarium udum* and NaCl stresses by enhancing expression of stress responsive *CcWRKY* genes	Kumar et al. (2019)
	PGPB + humic acid	Heat	*S. lycopersicum*	Pot experiments	Improved biomass, chlorophyll and salicylic acid content, antioxidant enzymes activities, Fe, P, and K uptake, augment heat stress response, reduce *SlWRKY33b* and *SlATG5* expression	Khan et al. (2020)

## Conclusion and future prospective

The current research on biostimulants has received unprecedented attention in both academia and industry. As mentioned above, biostimulants have been globally used in food, vegetables, fruit trees, flowers, nurseries, etc., to achieve high agricultural productivity and reduce the consequences/adverse effects of climate change and agrochemicals. The rapid development of research and application of biostimulants contributes significantly to sustainable agricultural practices. Although the efficiency of biostimulants has been widely recognized and the prospects for development are promising, there are still many problems to be solved, as follows:

1. Biostimulant product specification and standardization. There are a wide variety of biostimulants, and the market products are mixed and uneven. As they are different from traditional chemical pesticides and fertilizers, it is urgent for relevant national departments and industries to formulate relevant laws, regulations, policies, regulations, and technical standards/frameworks or guidelines. Moreover, a sustainable production system of biostimulants must be ensured. Despite the natural origin of most of the biostimulants (e.g., by-products of different value chains, natural and cheap resources) the production process must respect the circular economy and sustainability concept.
2. Lack of efficient production technology. Although all types of biostimulant products are currently produced and sold worldwide, most of them are primary products, and only a few high-end products are in the market as there is a lack of preparation technology for high-quality products (such as high purity and high activity) or the failure to achieve industrial application/implementation. Superior scientific research units and enterprises should speed up technological innovation in this field, deepen industry-university-research unit cooperation, and solve technical problems for further efficient production. For instance, more research on efficient techniques to improve and enlarge the shelf-life of most biostimulants is crucial.
3. The mechanism of action is still unclear, particularly under stress conditions. Due to the relatively complex composition of biostimulants, this feature determines that the target of its action mechanism is not very clear. Thus, the research on its action mechanism can be a long and complicated process. Some of the benefits demonstrated in plants growing under optimal conditions are not verified under stress conditions. Selecting a single compound with strong activity and clear structure from specific biostimulants to study the mechanism of action under specific environmental conditions (e.g., drought, salinity and extreme temperatures) could be a better model for in-depth research in this area and increase the knowledge of the biostimulants effects under climate change scenarios.
4. The application technologies are still largely unrealized. The concept of biostimulants comes from practical applications, however, there are also problems with specific application technologies. Through field trials, technology popularization, and application, it is an important guarantee for its industrial application to clarify the use of various biostimulants for a variety of crops and diverse conditions in different regions considering climate change scenarios. This requires normative experiments and regular summaries from scientific researchers and related practitioners.

All the mentioned problems will be gradually solved under the background of public needs and incentives, national requirements, and the attention of the industry. Recently, the research on biostimulants has become a hot spot in the field of plant protection and the use of biostimulants as an alternative to pesticides and fertilizers plays an important role in developing sustainable agriculture. In addition, all future improvements in the application of biostimulants for modern agriculture, e.g., the development of new products and/or upgrades, production, marketing and distribution of commercial biostimulants, could implement a sustainable strategy to improve plant tolerance/resistance against such environmental limitations, which is of great importance to secure and optimize global agricultural production under climate change scenarios.

## Author contributions

YM developed the ideas and wrote and revised the manuscript. MD wrote and revised the manuscript. HF was the project sponsor, and revised the manuscript. All authors contributed to the article and approved the submitted version.
